# Graft ischemia post cell transplantation to the brain: Glucose deprivation as the primary driver of rapid cell death

**DOI:** 10.1016/j.neurot.2024.e00518

**Published:** 2025-01-09

**Authors:** Abrar Hakami, Sebastiano Antonio Rizzo, Oliver J.M. Bartley, Rachel Hills, Sophie V. Precious, Timothy Ostler, Marija Fjodorova, Majed Alghamdi, Anne E. Rosser, Emma L. Lane, Thomas E. Woolley, Mariah J. Lelos, Ben Newland

**Affiliations:** a School of Pharmacy and Pharmaceutical Sciences, Cardiff University, King Edward VII Avenue, Cardiff, CF10 3NB, UK; b Department of Pharmaceutics, Faculty of Pharmacy, King Abdulaziz University, Jeddah 21589, Saudi Arabia; c Brain Repair Group, School of Biosciences, Life Sciences Building, Cardiff University, Cardiff, CF10 3AX, UK; d Cardiff School of Mathematics, Cardiff University, Senghennydd Road, Cardiff, CF24 4AG, UK; e Neuroscience and Mental Health Research Institute, School of Medicine, Cardiff University, Cardiff, CF14 4XN, UK; f Brain Repair and Intracranial Neurotherapeutics (B.R.A.I.N.) Biomedical Research Unit, College of Biomedical and Life Sciences, Cardiff University, Cardiff, UK; g Leibniz-Institut für Polymerforschung Dresden, Max Bergmann Center of Biomaterials Dresden, Hohe Straße 6, D-01069 Dresden, Germany

**Keywords:** Cell transplantation, Ischemia, Hypoxia, Glucose, Cell survival

## Abstract

Replacing cells lost during the progression of neurodegenerative disorders holds potential as a therapeutic strategy. Unfortunately, the majority of cells die post-transplantation, which creates logistical and biological challenges for cell therapy approaches. The cause of cell death is likely to be multifactorial in nature but has previously been correlated with hypoxia in the graft core. Here we use mathematical modelling to highlight that grafted cells experiencing hypoxia will also face a rapid decline in glucose availability. Interestingly, three neuron progenitor types derived from stem cell sources, and primary human fetal ventral mesencephalic (VM) cells all remained highly viable in severe hypoxia (0.1 ​% oxygen), countering the idea of rapid hypoxia-induced death in grafts. However, we demonstrate that glucose deprivation, not a paucity of oxygen, was a driver of rapid cell death, which was compounded in ischemic conditions of both oxygen and glucose deprivation. Supplementation of glucose to rat embryonic VM cells transplanted to the adult rat brain failed to improve survival at the dose administered and highlighted the problems of using osmotic minipumps in assisting neural grafting. The data shows that maintaining sufficient glucose in grafts is likely to be of critical importance for cell survival, but better means of achieving sustained glucose delivery is required.

## Graphical abstract

**Figure:**
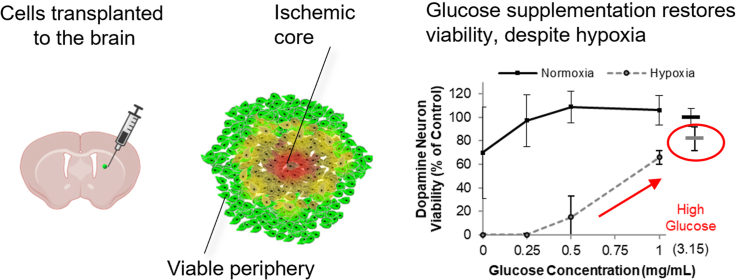

## Introduction

Cell transplantation offers a potential therapy for neurodegenerative diseases by replacing the cell populations that have degenerated due to disease, thereby enabling functional restoration of these neurons and reversal of the progression of the disease [1]. Clinical trials are underway worldwide to test the safety and efficacy of this approach for Parkinson's disease [2] and epilepsy [3], with other cell therapies in development for many other diseases [4,5]. However, typically less than 10 ​% of the grafted cells survive post-transplantation to the brain [6,7]. Attempts to circumvent this problem include grafting large numbers of neurons to compensate for the inevitable loss (for example, in Parkinson's disease, grafting ∼4 million cells to achieve the ∼100,000 remaining dopamine cells required [8]). However, this compensatory approach is not without drawbacks. Grafting larger numbers of neurons than required, but expecting the majority to die, will inevitably result in a larger physical space occupied initially by the graft (causing host tissue damage). Furthermore, the higher antigen load, as well as the presence of dying cells/cell debris, could exacerbate the host inflammatory response. This, in turn, may create a sub-optimal environment for maturation/integration of critical progenitor populations (to which dopamine neurons are particularly vulnerable [9]), leading to varying outcomes in terms of graft population and functionality [10]. Lastly, logistical challenges include the requirement to scale up manufacturing to generate large numbers of stem cell-derived neurons *in vitro*, and the impact on the surgical parameters (e.g. longer infusion times elongates surgery sessions in vulnerable patients). Current pre-clinical studies therefore fall short of their potential efficacy, with overcompensation at administration not addressing the root cause of the problem.

The majority of cell loss in grafts occurs within the first week, with as much as 80 ​% in the first 24 ​h [11]. Whilst the cause of cell death post-transplantation is likely to be multifactorial, Praet et al. showed that cells experience extreme hypoxia within hours of post-transplantation into the brain, especially in the core of the graft [12]. Since the hypoxic regions in these grafts correlated with apoptosis and cell loss, we previously predicted that oxygen-producing microspheres could improve graft survival [13]. However, upon *in vitro* analysis, we determined that, if glucose was present in the medium, a variety of cell types could survive prolonged extreme hypoxia without loss of viability. We therefore suggested that the hypoxia-apoptosis correlation was not causal, but perhaps indicative of a general lack of nutrients in the graft core during the early stages post-transplantation.

Petite and co-workers have shown that the survival of mesenchymal stem cells is heavily dependent on glucose rather than oxygen [[14], [15], [16]]. They suggested that the hypoxia seen in the graft core is due to inadequate vascularization/blood perfusion and accompanied by a lack of glucose. The graft site therefore represents a condition of oxygen and glucose deprivation (OGD), which might be responsible for the massive cell loss seen within the first days post-transplantation. Furthermore, we reviewed oxygen-glucose deprivation studies on neural cells and highlighted the significance of glucose in the maintenance of cell viability [17].

Herein we sought to investigate the role that glucose plays in the survival of neuron progenitors subjected to conditions that they may face in a neural graft. First, mathematical modelling was used to predict the degree and time course of ischemia in a deposit of grafted cells. Then, we exposed three different neuron progenitor populations to extreme hypoxia and varying degrees of glucose deprivation to assess empirically the relative impact of oxygen vs glucose deprivation. Finally, we conducted an animal study to examine the ability of osmotic mini pumps to provide exogenous glucose to grafted fetal ventral mesencephalic (fVM) cells and improve their survival in the early stages post-transplantation in a rat model of Parkinson's disease.

## Materials and Methods

### Modelling oxygen and glucose depletion in grafted cells

**Fig. 1:**
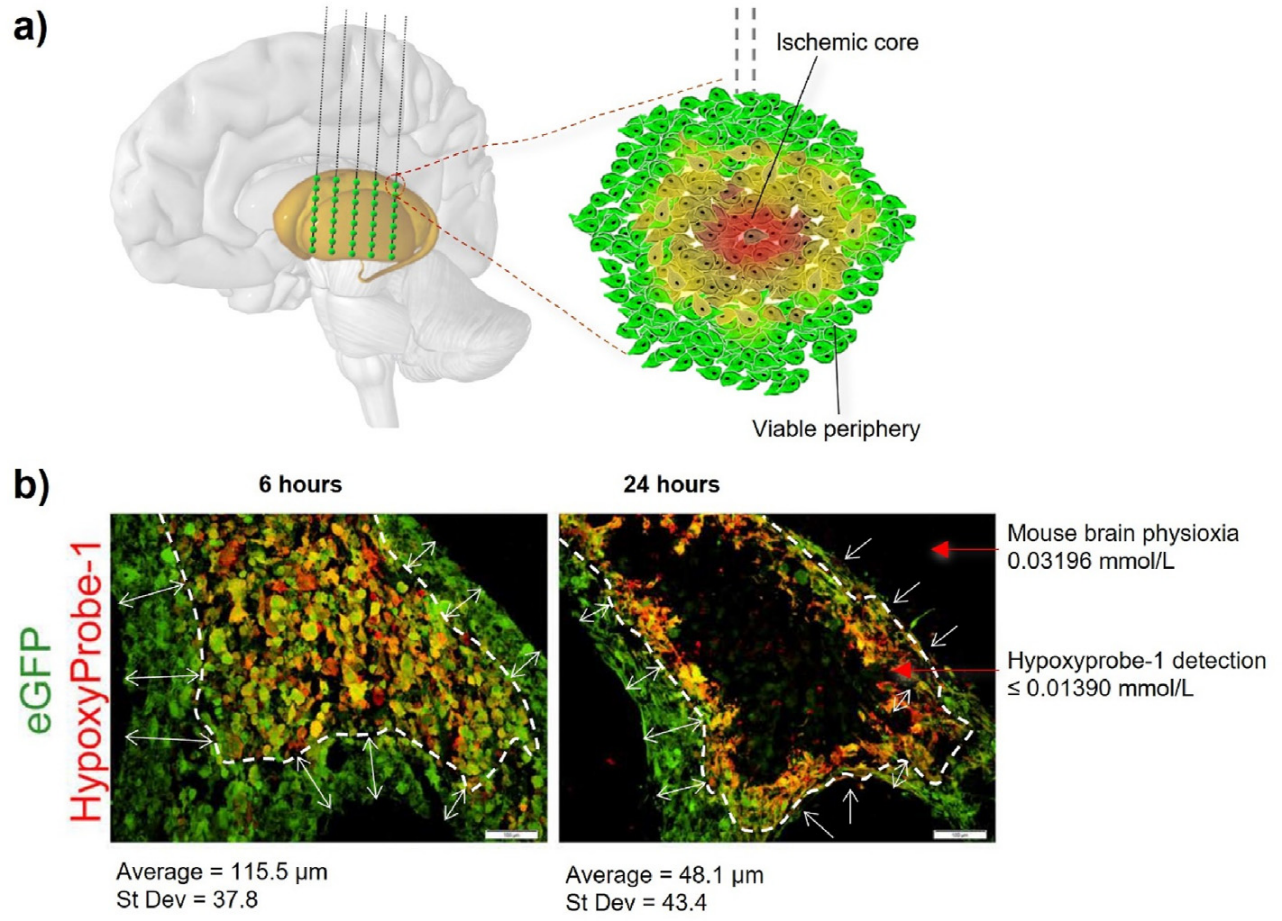
Ischemia may be the primary cause of cell death post-transplantation to the brain. (A) A schematic depiction of multiple tracts and multiple graft deposits required for cell transplantation therapy in the striatum, showing our hypothesis that there is a gradient of nutrient paucity in each graft deposit. (B) Data from Praet et al. was used to estimate the distance from the graft periphery at which hypoxia is present at 6- and 24-h post-transplantation (image used with permission from Ref. [12]).

Dimensions for a graft deposit size used for the modelling computations were determined using the latest recommendations of the TRANSEURO consortium [8]. These indicate that five tracts, each with eight deposits of 2.5 ​μL would be the most likely optimal approach to provide functional recovery based on analysis of previous surgical approaches (Fig. 1A). (i.e., 100 ​μL transplanted in total). Although no starting number of cells was stated, calculations were based upon a plausible use of 100,000 ​cells per μL, so 250,000 ​cells per deposit. The average diameter of a cell is 9.1 ​μm as taken from images of cells lifted from their substrate. Assuming that it is a sphere its volume would be 4/3 ​× ​π ​× ​(9.1/2)^3^ ​≈ ​395 ​μm^3^. It is estimated that there are around 250,000 ​cells in a graft, giving the graft a volume somewhere between 250,000 ​× ​395 ​μm^3^ ​≈ ​9.9 ​× ​10^7^ ​μm^3^ and 250,000 ​× ​395/0.64 ​μm^3^ ​≈ ​15.4 ​× ​10^7^ ​μm^3^ where the factor of 0.64 comes as the sphere packing coefficient since spheres do not pack perfectly, as there are points of empty space. Thus, if the graft is also a sphere, then the radius of the equivalent sphere would be between 287 and 333 ​μm. Due to this variability, we choose to consider a graft of radius 300 ​μm as a means of picking a simple order of magnitude size.

Data from Praet et al. [12] was used to approximate the distance from the graft periphery at which hypoxia is present at 6- and 24-h post-transplantation (Fig. 1B). This data was used to determine the diffusion coefficient of oxygen, assuming that regions positive for HypoxyProbe-1 staining contained oxygen at less than or equal to 0.01390 ​mmol/L (manufacturer's information). We assume that both oxygen and glucose diffuse from outside the graft to the inside. Moreover, we assume that the oxygen and glucose are in excess outside of the graft and, thus, their concentrations can be considered fixed at values 0.03196 ​mmol/L [18] and 1.66 ​mmol/L [19], respectively, on the boundary of the spherical graft. Alongside their ability to diffuse within the graft the oxygen is used up at a constant rate, γ_*O*_. Equally, the consumption rate of glucose, γ_*G*_(*O*), is a piecewise constant, depending on the local concentration of oxygen. Namely, when the concentration of oxygen is above 0.0139 ​mmol/L (normoxia), the consumption rate of glucose is 0.3 mmol/(L ⋅ min), whilst if the concentration of oxygen is below 0.0139 ​mmol/L (hypoxia), the consumption rate of glucose is 1.6 mmol/(L ⋅ min), as determined by Wohnsland et al. *in vitro* [20].

Abstracting these ideas into mathematics we model the system using two diffusion equations in three-dimensional spherical polar coordinates [21],

(1) $$\frac{\partial O}{\partial t}=\frac{D_{O}}{r^{2}}\frac{\partial }{\partial r}\left(r^{2}\frac{\partial O}{\partial r}\right)-\gamma_{O,}$$

(2) $$\frac{\partial G}{\partial t}=\frac{D_{G}}{r^{2}}\frac{\partial }{\partial r}\left(r^{2}\frac{\partial G}{\partial r}\right)-\gamma_{G}\left(O\right)\text{,}$$

where *O*(*r*,*t*) and *G*(*r*,*t*) represent the concentrations of oxygen and glucose, respectively, at a time *t* and spatial distance *r* from the centre of the graft. Further, *D*_0_ and *D*_*G*_ represent the diffusion rates of oxygen and glucose, respectively. From the literature *D*_0_ is of the order 10^3^μ m^2^/s, whilst *D*_*G*_ is approximately 5 times smaller as it is a larger molecule and, thus, diffuses slower [[22], [23], [24]]. Initially, the concentration of oxygen and glucose are constant throughout the grafts and are set to their boundary values of *O*(300,*t*) ​= ​0.03196 ​mmol/L and *G*(300,*t*) ​= ​1.66 ​mmol/L. We solve the equations over a simulation period of 24 ​h to determine the level of glucose in the graft.

### *In vitro* analysis

#### Culture and differentiation of human pluripotent stem cells (PSC) hPSC culture

hPSC derived neurons were derived from hESC lines H9mChry (WIZ04e-H9CAGmChry; WiCell), iCr-hESC (González et al., 2014), and human fetal LGE derived hiPSC line L2 (Bartley et al., 2024). Prior to differentiation, hPSCs were cultured in Essential 8 Flex medium (E8; Gibco) on Matrigel (Corning) coated plates, at 37 ​°C in 5 ​% CO_2_. Medium was changed every other day. hPSCs were passaged at ∼60–80 ​% confluency, using EDTA (Gibco).

#### Dopaminergic neuron (DA) differentiation

DAs were differentiated as previously described (Jaeger et al., 2011). In brief, hPSCs were passaged onto plates pre-treated with Growth Factor Reduced Matrigel (GFR Matrigel, Corning, 1:15 dilution in DMEM/F-12) and cultured in E8 Flex medium until ∼80 ​% confluent. DA differentiation was initiated by replacing E8 Flex medium with N2B27-RA medium (DMEM-F-12/Neurobasal media (2:1) supplemented with l-Glutamine (1:100), N2 (1:150), and retinol-free B27 (1:150) β-mercaptoethanol (1:1000) (all Gibco), and either Pen/Strep (1:1000, Gibco) or Mycozap (1:500, Lonza). For the first 7 days, N2B27-RA medium was further supplemented with 100 ​nM LDN-193189 (LDN, StemGene), 200 ​nM Dorsomorphin (DM, R&D) and 10 ​μM SB431542 (SB, Tocris). From day 3, N2B27-RA was further supplemented with 1 ​μM PD03 (Axon). From day 7, previous supplemented factors were removed from N2B27-RA, and medium was instead supplemented with 200 ​ng/mL hShh (PeproTech) and 1 ​μM purmophamine (PURM, PeproTech). Neural precursors were passaged between day 9–10, by treating for 1 ​h with 10 ​μM Y27632 (Millipore), and then passaging using EDTA onto Fibronectin (Millipore) and PDL (Gibco) treated plates at a 2:3 ratio. N2B27-RA was supplemented with hShh, purmophamine, and 100 ​ng/mL FGF8 (PeproTech). On day 16, N2B27-RA was changed to N2B27 (retinol-free B27 replaced with B27), and supplemented with 10 ​ng/mL BDNF, 10 ​ng/mL GDNF (both PeproTech), and 0.2 ​mM l-Ascorbic Acid (Sigma). DA progenitors were passaged on day 22, and frozen in 20 ​% DMSO for long term storage. Throughout differentiation, culture medium was changed every other day.

#### Medium spiny neuron (MSN) differentiation

MSNs were differentiated as previously described (Arber et al., 2015). In brief, hPSCs were passaged onto plates pre-treated RGF Matrigel and cultured in E8 Flex medium until ∼80 ​% confluent. MSN differentiation was initiated by replacing E8 Flex medium with N2B27-RA medium, supplemented with LDN, Dorsomorphin, and SB. SB was excluded from medium after 5 days. Between days 9–10 neural precursors were passaged by treating for 1 ​h with Y27632, and then passaging using EDTA onto Fibronectin and PDL treated plates at a 2:3 split ratio. From day 9, N2B27-RA was instead supplemented with 25 ​ng/mL Activin A (R&D). MSN progenitors were passaged between day 18–20, and frozen in 20 ​% DMSO for long term storage. Throughout differentiation, culture medium was changed every other day.

#### Glutamatergic projection neuron (GPN) differentiation

Glutamatergic projection neurons (GPNs) were differentiated as described for MSNs, but without Activin A supplementation from day 9.

#### Neuron progenitor characterization

The neuron progenitors were characterized as reported/quantified in a previous publication [25]. Immunocytochemical analysis of the three neuron types is show in Supplementary Fig. S1, with DA neurons visualized via tyrosine hydroxylase (TH) immunostaining, MSNs via dopamine- and cAMP-regulated neuronal phosphoprotein (DARPP-32) and COUP-TF-interacting protein 2 (CTIP2), and GPNs via T-box, brain, 1 (TBR1).

#### Defrosting and preparation for glucose deprivation

Four days prior to experimental procedures, hPSC derived neuronal progenitors (DA, MSN and GPN) were thawed using an automated cell thawing platform (ThawSTAR, Biocision), and plated in N2B27 medium at 20–30,000 ​cells per well of 96 well flat-bottomed culture treated plates, coated with PDL and laminin (Lonza), at 37 ​°C in 5 ​% CO_2_. N2B27 was supplemented with BDNF and GDNF. Medium was changed every other day.

#### Collection and culture of human fetal tissue

Human fVM (hVM) tissue was acquired from the SWIFT (South Wales Initiative for Fetal Tissue) tissue bank, under the UK Human Tissue Authority research license held by Cardiff University (No. 12422). Gestational age was estimated using measurements taken at the time of dissection [26]. Following dissection, hVM tissue was stored in Hibernate-E medium (Gibco) for 3 days prior to dissociation. For dissociation and plating, hVM was incubated at 37 ​°C for 20 ​min in TrypLE Express (Gibco) with 20 U/mL dornase alfa (Gentech), then dissociated by trituration in DMEM/F-12 with 20 U/mL dornase alfa. Dissociated hVM cells were plated in NDM medium (DMEM/F12, FCS (1:100), B27 (1:100), and Pen/Strep (1:100) (all Gibco)) at 25,000 ​cells per well of 96-well flat-bottomed culture treated plates, coated with PDL and laminin. hVM derived cells were cultured in NDM medium at 37 ​°C in 5 ​% CO_2_ for 9 days prior to further experimental procedures. At day 0 of the OGD experiment, culture medium was replaced with 100 ​μL of fresh NDM (normal medium control) or SILAC DMEM/F-12 with either no glucose or glucose at a final concentration of 1, 0.5 and 0.25 ​mg/mL. Plates were then incubated either in normoxia (37 ​°C, 21 ​% O_2_ and 5 ​% CO_2_) or in near anoxia (37 ​°C, 0.1 ​% O_2_, 5 ​% CO_2_ and 94.9 ​% N_2_) in a BioSpherix ProOx C21 hypoxia chamber. At day 1 and day 3, morphological changes were evaluated with a brightfield microscope (EVOS M7000, Thermofisher) and cell viability was assessed with the PrestoBlue assay (Thermofisher) according to the manufacturer's protocol as detailed below.

#### Cell culture for the study of glucose and oxygen deprivation

Cells were cultured in mediums suitable for their cell type as described above. For hPSC derived DA/MSN/GPN neurons this comprised of N2B27 medium supplemented with GDNF and BDNF, and for VM cells this was NDM. This is represented in the graphs as “Normal Medium” (NM) and contains 3.15 ​mg/mL of glucose. However, to create medium with reduced glucose (0–1 ​mg/mL), DMEM/F-12 was replaced with SILAC Advanced DMEM/F-12 (Gibco) and Neurobasal was replaced with Neurobasal-A, no d-glucose, no sodium pyruvate (Gibco). These mediums were then supplemented with glucose (Fisher Scientific) at concentrations ranging from 0 to 1 ​mg/mL. Cell cultures were either maintained in normoxic (20 ​% oxygen) or hypoxic conditions (0.1 ​% oxygen in a BioSpherix ProOx C21 hypoxia chamber) for the duration of the experiment.

#### Analysis of cell viability in various levels of oxygen and glucose

Cell viability was determined using the PrestoBlue cell viability reagent (Thermofisher):10 ​% (v/v) PrestoBlue was added to culture media, and cell cultures were returned to present culture conditions for up to 5 ​h. The fluorescence signal was measured using either a FLUOstar Optima (BMG Labtech) or an Infinite 200 Pro (Tecan) plate readers (excitation/emission 560/590 ​nm). Values were normalized to control wells in normoxic conditions in normal medium.

### *In vivo* analysis of grafted VM cells after exogenous glucose delivery

**Fig. 2:**
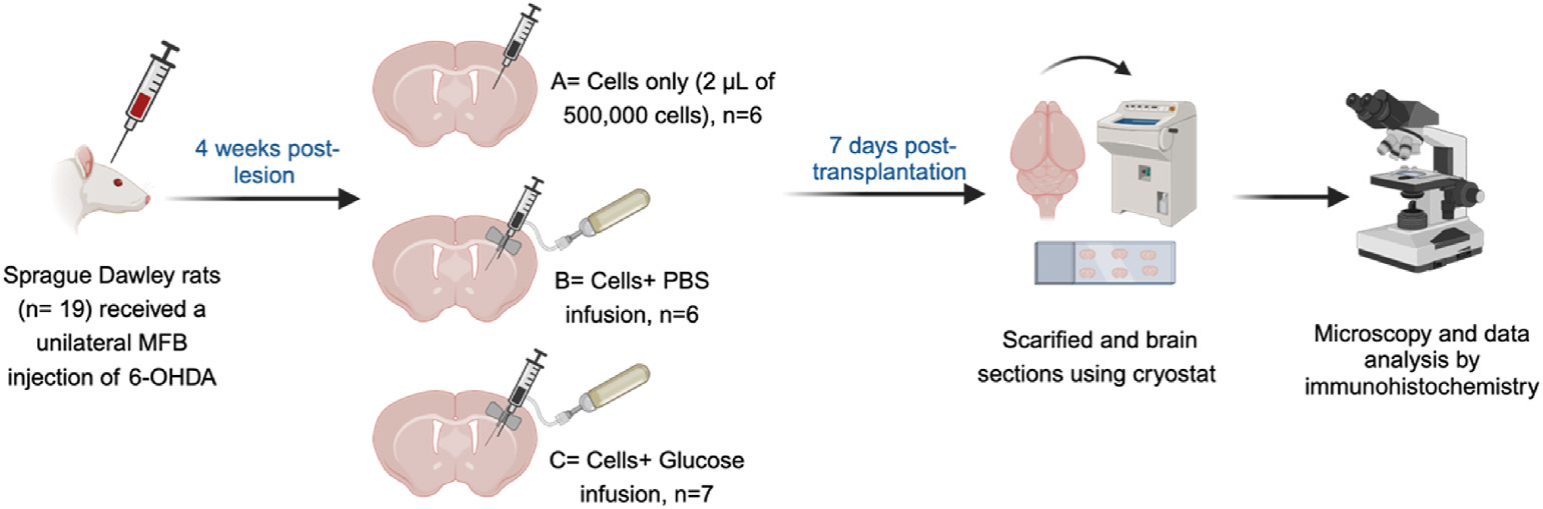
Experimental design for biological studies *in vivo*. 19 Sprague Dawley rats received unilateral median forebrain bundle (MFB) injection of 6-OHDA then rats were randomly divided into three groups, A-cells alone (negative control), B- cells with pump containing a saline solution (control), and C- cells with pump containing glucose (treatment), created with BioRender.

An *in vivo* study was carried out to determine the effect of an osmotic mini pump providing a continuous supply of either phosphate buffered saline (PBS) or glucose to the graft site. We aimed to investigate both the host immune response and whether the provision of glucose can prevent the extensive cell death of transplanted cells (Fig. 2). 6-Hydroxydopmamine (6-OHDA) lesioned rats were randomly assigned to receive either VM cells from E14 donors alone or along with PBS or glucose infusion. Rats were sacrificed at 7 days post-transplantation then the brains were sectioned and collected in a 1:12 series for immunohistochemical analysis of dopaminergic neurons and microglia/astrocyte responses as outlined below.

#### Study protocol

20 Sprague Dawley rats were purchased from Charles River (Margate, UK) and housed under conditions of 12 ​h light/12 ​h dark (lights on at 06.00 ​h) with water available ad libitum. Rats were fed crushed diet [SDS 801066 RM3(E); 3.64 ​kcal/g)] which was available ad libitum, with food intake and body weight being monitored. Before the study could commence, 1 rat was euthanized due to an ear infection, hence leaving 19 candidates. The animals were anaesthetized using 5 ​% isoflurane in oxygen as a carrier gas for induction to a surgical plane, followed by maintenance at 2.5 ​% isoflurane. The rodents were administered with a single unilateral median forebrain bundle (MFB) injection of 6-hydroxydopmaine (hydrobromide salt (Sigma Chemicals, UK); 3 ​μg/μL in vehicle). The solution was injected into the right MFB using the following co-ordinates (relative to Bregma) Lateral ​− ​1.3 ​mm; Anterior ​− ​4.0 ​mm and Ventral 7.0 ​mm. A total volume of 3 ​μL 6-OHDA was injected at a flow rate of 1 ​μL/min, the syringe was left in place for a further 3 ​min and then slowly removed. The rats received an injection of analgesic (Metacam, 150 ​μg/s.c.) after the skin was sutured and they were allowed to recover before being returned to their home caging.

All rats received grafts of rat VM cells from E14 donors (prepared as previously described [27]), 4 weeks post-lesion but were randomly assigned to the following groups: A - cells only (negative control) (n ​= ​6), B - cells with an infusion of phosphate buffered saline (PBS) (cells ​+ ​PBS) (n ​= ​6) and (C) cells with an infusion of a glucose solution in PBS (cells ​+ ​glucose) (n ​= ​7). All rats received a total volume of 2 ​μL (total of 500,000 ​cells) at a rate a 1 ​μL/min using the following co-ordinates from Bregma and dura: AP: +0.6, ML: −2.6, DV: −6.0 and tooth bar set to −3.2. Immediately after transplantation, the graft groups B and C received osmotic minipumps (Alzet 1004 Pump #10104846)) in conjunction with the Brain Infusion Kit (BIK) (# 10104827), placed so that the cannula sits directly above the graft site. Pumps were either loaded with sterile PBS, or with glucose in PBS at a concentration of 38 ​mg/mL, with a flow rate of 0.11 ​μL per hour, thus continuously delivering 0.1 ​mg per day to the 500,000 transplanted cells. 7 days post-transplantation the rats were ethically terminally anaesthetized. The transcardial perfusion was done with ∼150 ​mL PBS, followed by ∼250 ​mL of refrigerated 4 ​% paraformaldehyde (PFA). After that, brains were post-fixed in PFA for 4 ​h post-extraction, then transferred to 25 ​% sucrose in PBS solution. Coronal sections (20 ​μm) were cut along the entire rostro-caudal extent of the mid-brain using a cryostat (Cryotome FSE, Fisher Scientific) and collected in 1:12 series, which were placed directly onto microscope slides (SuperFrost Plus Adhesion slides, Fisher Scientific, #12625336) ready for immunohistochemical analysis.

#### Immunohistochemistry

Slides with serial coronal sections (1:12) were used for immunohistochemistry for dopaminergic neurons (tyrosine hydroxylase, TH), microgliosis (cd11b, OX 42) and astrocytosis (glial fibrillary acidic protein, GFAP).

Slide mounted sections were placed in a water bath with pH ​= ​6 of citric acid at 70 ​°C for 30 ​min for antigen retrieval. Slides were removed from the water bath to cool. A hydrophobic pen was used to mark around brain sections, keeping subsequent solutions on and around tissue without escaping. Tris-buffered saline concentrated stock solution (X4 TBS) was prepared containing 48 ​g TRIS base, 36 ​g sodium chloride and distilled water 1000 ​mL then the pH was adjusted to 7.3 with concentrated HCl and stored at 4 ​°C. 0.1 ​M TBS working solution was prepared by taking 250 ​mL of stock solution and 750 ​mL of distilled water and the pH was adjusted to 7.4 with concentrated NaOH or HCl and stored at 4 ​°C. Slides were submerged in TBS (3 ​× ​10 ​min), TBS was used as a washing buffer. After that, sections were quenched to inhibit endogenous peroxidase activity in 6.25 ​mL methanol, 6.25 ​mL hydrogen peroxide (H_2_O_2_) and 40 ​mL distilled water for 5 ​min. Slides were washed (TBS; 3 ​× ​10 ​min) and blocked for 1 ​h using 3 ​% goat serum (TH) or horse serum (cd11b and GFAP) in 0.2 ​% Triton X-100 in TBS (TxTBS) solution. Triton X-100 solubilizes proteins and so allows antibodies to penetrate through the cell. Then, slides were transferred, without washing, into primary antibody in TxTBS with 1 ​% serum. Primary antibody use was as follows; for TH cells; Rabbit anti-tyrosine hydroxylase antibody (AB152, Merck, 1:2000), for microglia cells; Mouse anti-CD11b antibody (MCA275G, Serotec, 1:3000) and for astroglia cells; Mouse anti-GFAP antibody (ab279290, abcam 1:50). Parafilm was placed over slides and incubated overnight. Slides were washed (TBS 3 ​× ​10 ​min) and placed in a biotinylated secondary antibody for 3 ​h (either goat anti-rabbit IgG biotinylated (BA1000, Vector Labs, 1:200) for TH staining or horse anti-mouse IgG biotinylated (BA2001, Vector labs, 1:200) for CD11b and GFAP staining). Next, slides were washed (TBS 3 ​× ​10 ​min) and incubated in Avidin-Biotin Complex (ABC) solution for 2 ​h. ABC was prepared 30 ​min before use and left to mix. Then, slides were washed (TBS 3 ​× ​10 ​min) in 0.05 ​M Tris non-saline (TNS) solution containing TRIS base 6.0 ​g in 1000 ​mL distilled water. Finally, 5 ​% solution of diaminobenzidine tetrahydrochloride (DAB, Sigma) containing 0.3 ​μL/mL of hydrogen peroxide in TNS solution was prepared (40 ​mL TNS, 2 ​mL DAB solution and 12 ​μL ​H_2_O_2_) and pipetted over slides for 30 ​min, then washed with (TBS 3 ​× ​10 ​min). All slides were submerged in alcohol from low to high concentration 70, 95, 100 ​% then were submerge in xylene to dehydrate. Finally, slides were cover slipped using DPX mountant (# 06522, Merck).

#### Data analysis

All measurements were estimated using unbiased stereology. Two-dimensional stereology was performed with Zeiss Axio scan Z1 slide scanner and was processed using Zeiss ZEN blue 3.6 software (Zen Research Microscopy Solutions) and FIJI ImageJ (NIH) for optical density. All quantitative immunohistochemistry data was analyzed from cross-sectional areas measured on a 1:12 series of sections throughout the rostro caudal axis of the striatum and was completed by three people independently, all blinded to the treatment of the rats.

o *For Dopamine neurons:*

##### TH counting

Dopamine neurons in the processed brain tissue were identified via TH staining. The number of surviving dopamine cells was calculated by counting all TH positive cells visible in the sections and multiplying by 12 to get the whole number of TH cells of the graft for each rat (counted manually using ZEN Lite by three different people with the average value used in the analysis).

Estimated cell number ​= ​d ∗ b, where d is the sum of cells counted on each section and b is the frequency of sections (12).

##### Graft volume

The graft area was measured using cross-sectional areas from a 1:12 series of TH-stained sections for each rat. The volume was calculated from the following equation: volume (mm^3^) ​= ​a ∗ b ∗c/1,000, 000, 000. Where a is the sum of all areas, b is the frequency of sections (12), and c is the section thickness (20 ​μm).

##### TH cell density

The TH density was calculated by dividing the average cell number by the average graft volume for each rat.

Cell density (cells/mm^3^) ​= ​estimated cell number/volume.

##### Percentage survival of TH cells

TH cell survival was estimated by dividing TH^+^ counts by the number of total cells transplanted (500,000 per rat). It should be noted that these results are an estimation of the percentage of cell survival since we only identified TH^+^ dopamine neurons and no other cells which were also present in VM transplanted cells.

o *For the microglia and astrocyte response:*

The optical density of microgliosis and astrocytosis immunostaining were measured from a 1:12 series of sections. All sections in which microgliosis/astrocytosis expression were detected were used in the analysis. The images were analyzed for mean grey values using FIJI ImageJ software (NIH) and converted into optical density (arbitrary units) by applying the following formula: OD ​= ​log10 (255/mean grey value). The microglia/astrocytes volume was calculated the same way for the graft volume as above. It should be noted that this method of analysis gives an overall indication of host response but does not directly quantify the microglia and astrocytes cell numbers. Counting each cell manually was technically challenging due to the high density of the microgliosis and astrogliosis at the graft site.

### Statistical analysis

Unless otherwise specified, *in vitro* data are reported as mean ​± ​standard deviation (n ​= ​5) of a single (N ​= ​1) or triplicate (N ​= ​3) experiments. For statistical analysis of *in vitro* data, IBM SPSS Statistics 27 software was used, and an ANOVA was carried out to identify between-subject effects (overall effects of reduced ‘Oxygen’ and reduced ‘Glucose’ as individual factors, as well as the interaction between these factors). This was followed by Sidak pairwise analysis to determine differences between cell viability in hypoxia and normoxia for each glucose concentration. *In vivo* data are expressed as mean ​± ​standard error of the mean and were analyzed using GraphPad Prism using a one-way analysis of variance (ANOVA) with Tukey's post-hoc tests to calculate the statistical significance of all results comparing results between each group.

## Results

### Modelling oxygen and glucose depletion in grafted cells

A typical transplantation paradigm for PD, would involve multiple cell deposits along multiple putaminal tracts [8] and we hypothesize that in each deposit there will be a gradient of reduced nutrients towards the graft core. Analysis of existing data on oxygen depletion in grafts in the mouse brain from Praet et al., showed that hypoxia detected by HypoxyProbe-1 (as an oxygen concentration less than 14 ​μM - equivalent to a partial pressure pO_2_ ​= ​10 ​mm Hg ​at 37 ​°C) was present as early as 6 ​h post-grafting and cell loss was shown by 24 ​h.

**Fig. 3:**
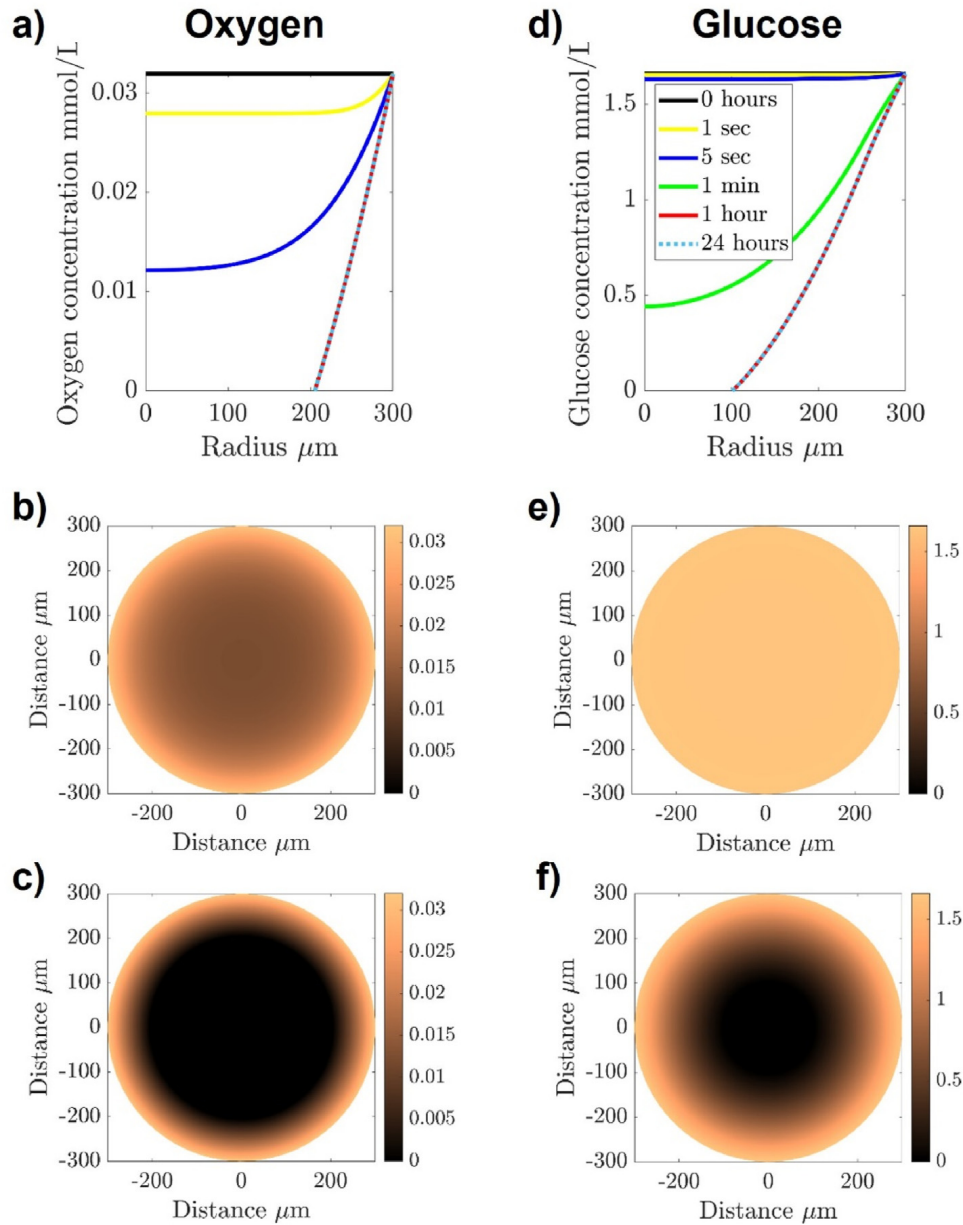
Mathematical modelling of oxygen (A–C) and glucose (D–F) depletion over 24 ​h in a graft deposit of 250,000 ​cells occupying a sphere of 300 ​μm radius once transplanted into the human brain. (A) The perimeter of the graft remains at physoxia throughout the first 24 ​h, but the oxygen concentration in the graft decreases towards the core in a time dependent manner as shown via heatmaps at 1 ​s (B) and 24 ​h (C). (D) A similar trend is shown for glucose, as shown in the heatmap at 1 ​s (E) but as the hypoxic core forms, the cellular shift to the less efficient glycolysis (and higher glucose consumption) compounds the problem of nutrient shortage over time, leading to a rapid drop-off in glucose concentration at the core by 24 ​h (F).

The boundaries of the hypoxic region at two time points as shown in the methods section were used to compute the oxygen diffusion coefficient through a typical graft. Mapping of the oxygen concentration in the grafted deposit of cells shows oxygen depletion in the graft core over the first 24 ​h (Fig. 3A–C). The paucity of oxygen then compounds the problem of glucose deficiency in the graft core, as cells switch to glycolysis for their energy needs, thus requiring additional glucose in the hypoxic core than that required at the periphery in physoxia. This results in a clear drop-off in glucose availability in the graft core (Fig. 3D–F).

### *In vitro* analysis to investigate the role of glucose in the survival of different neuron progenitor populations in OGD conditions

#### Calculation of graft glucose consumption during OGD

The results from the mathematical models further support the idea that glucose plays a pivotal role in graft survival. Moreover the graphical representation of the model also parallels the situation of ischemic strokes, with the presence of a necrotic/apoptotic core and a viable penumbra that has access to oxygen and glucose, which we previously hypothesized could represent the situation of grafted cells [28]. The glucose consumption rate in the case of grafted cells was calculated by using rates from Wohnsland et al., [29]. Considering 250,000 ​cells as an average number of transplanted cells, this calculation indicates a cumulative consumption of approx. 1.2 ​mg in 7 days under hypoxic conditions.

#### High availability of glucose is sufficient for neuronal progenitors to survive oxygen deprivation

**Fig. 5:**
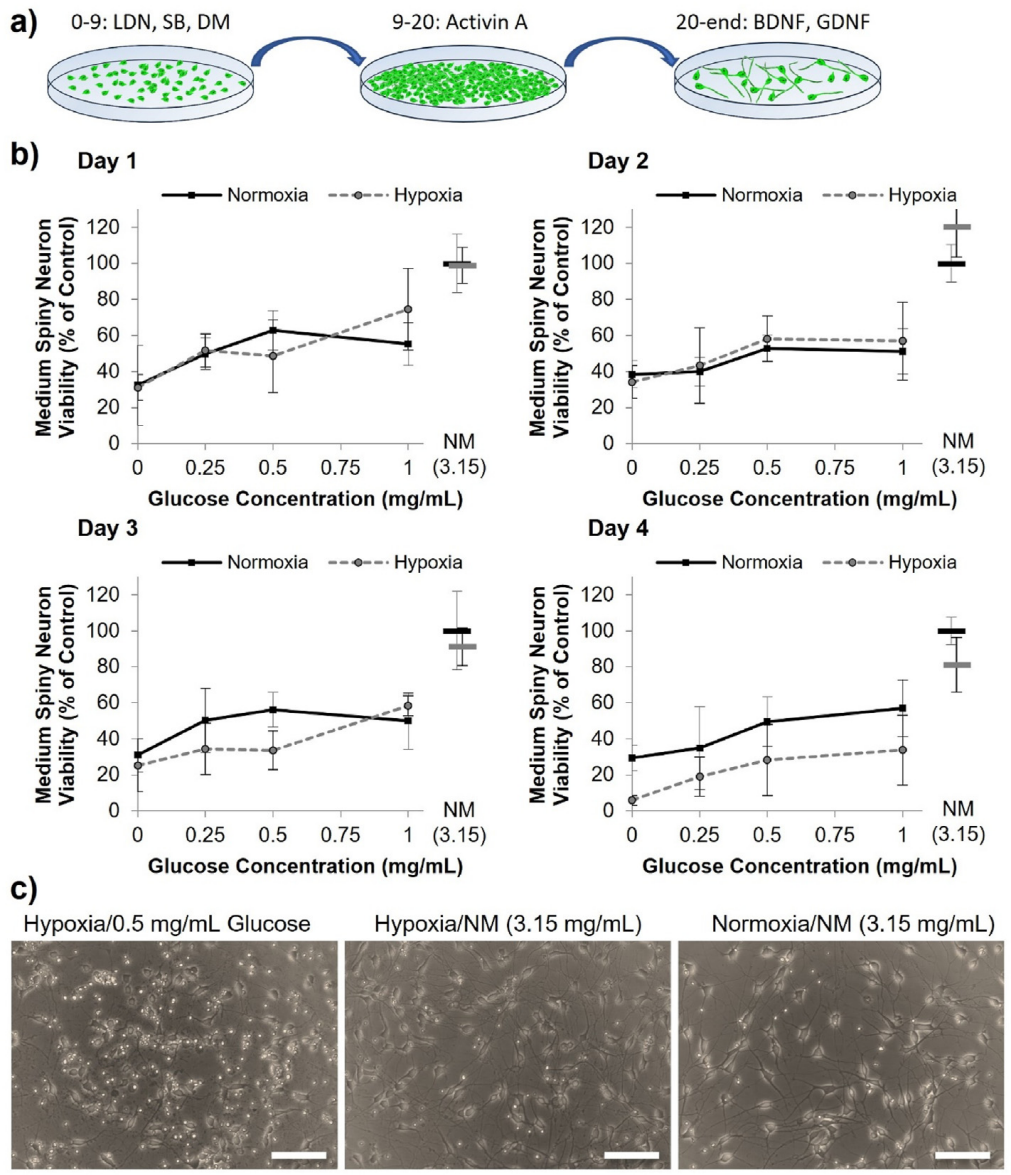
Stem cell-derived medium spiny neuron (MSN) progenitors suffer rapid, but modest loss in viability in oxygen-glucose deprivation conditions. (A) Overview of MSN progenitor differentiation. (B) The viability of MSN progenitor cultures, assessed using the PrestoBlue assay, following 1–4 days in normoxic (black, solid), or hypoxic (grey, dashed) conditions with different concentrations of glucose, where NM ​= ​normal medium containing 3.15 ​mg/mL. Data was normalized to normoxia normal media control (3.15 ​mg/mL glucose) (mean ​± ​SD, n ​= ​4). There was no statistically significant combinatorial effect of oxygen-glucose deprivation (i.e. no difference between the viability in hypoxia vs normoxia at any given glucose level. (C) Representative brightfield images of DA neurons at day 3 of culture in either hypoxic or normoxic conditions with 0.5 or 3.15 ​mg/mL glucose.

**Fig. 6:**
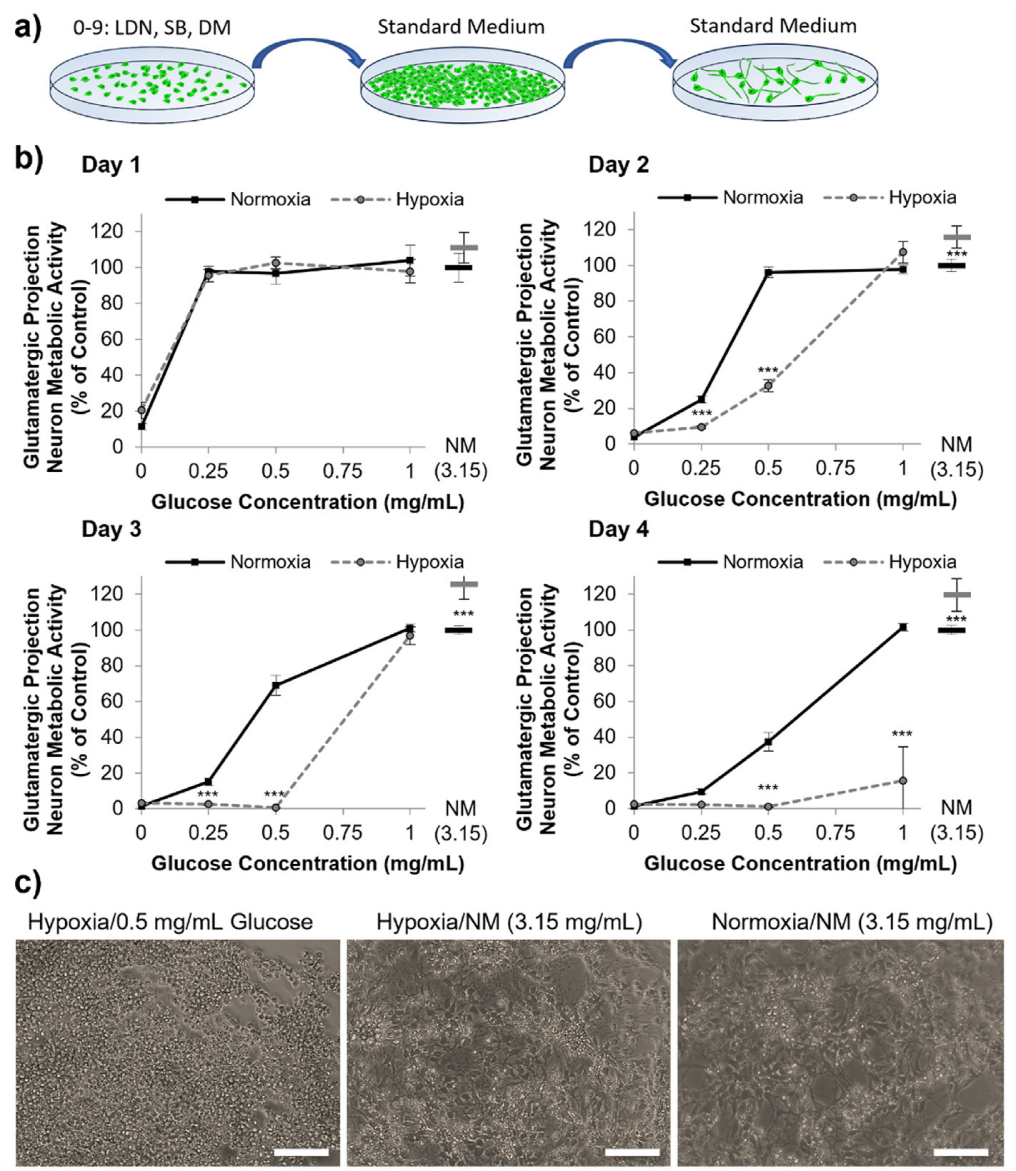
Glutamatergic projection neuron (GPN) progenitors lose viability rapidly and substantially in a paucity of glucose, which is exacerbated by hypoxia. (A) Overview of GPN progenitor differentiation; (B) The viability of GPN progenitor cultures, assessed using the PrestoBlue assay, following 1–4 days in normoxic (black, solid), or hypoxic (grey, dashed) conditions with different concentrations of glucose, where NM ​= ​normal medium containing 3.15 ​mg/mL **shown as stand-alone data points as bars (black** ​= ​**normoxia, grey** ​= ​**hypoxia)**. Data was normalized to normoxia normal media control (3.15 ​mg/mL glucose) (mean ​± ​SD, n ​= ​4). Asterisks show a statistically significant difference in viability in hypoxia compared to normoxia for a given glucose concentration, with hypoxia showing a reduction in viability at low glucose concentrations but an increase in viability in normal media and hypoxia for day 2–4 (∗∗p ​< ​0.01, ∗∗∗p ​< ​0.001). (C) Representative brightfield images of DA neurons at day 3 of culture in either hypoxic or normoxic conditions with 0.5 or 3.15 ​mg/mL glucose.

**Fig. 7:**
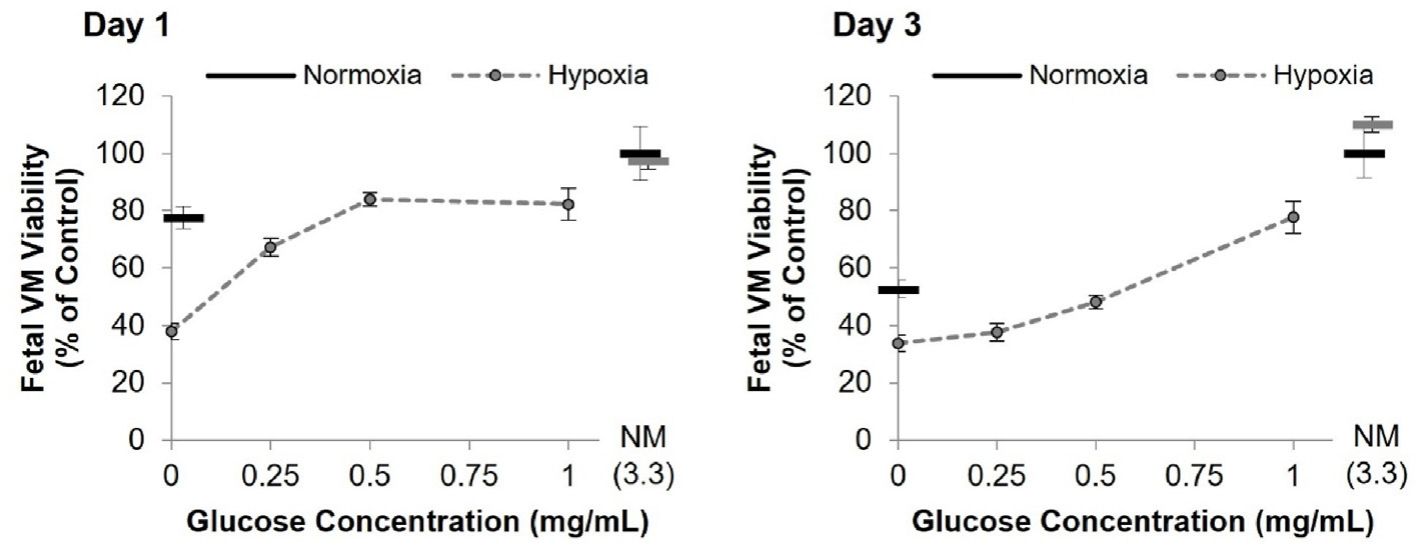
Primary human ventral mesencephalic (VM) progenitor cells were cultured either in normoxia (20 ​% oxygen, solid black bars) or near anoxia (0.1 ​% oxygen, dashed line) at different glucose concentrations. (A) The viability of hVM progenitor cultures, assessed using PrestoBlue assay, following 1–4 days in normoxic (black, solid), or hypoxic (grey, dashed) conditions with different concentrations of glucose, where NM ​= ​normal medium containing 3.3 ​mg/mL shown as stand-alone data points as bars (black ​= ​normoxia, grey ​= ​hypoxia). Data was normalized to normoxia normal media control (3.3 ​mg/mL glucose) (mean ​± ​SD, n ​= ​4). Due to the scarcity of the tissue, the full glucose dose response was performed only in near anoxia, while the normoxia plate were used as a control.

**Fig. 8:**
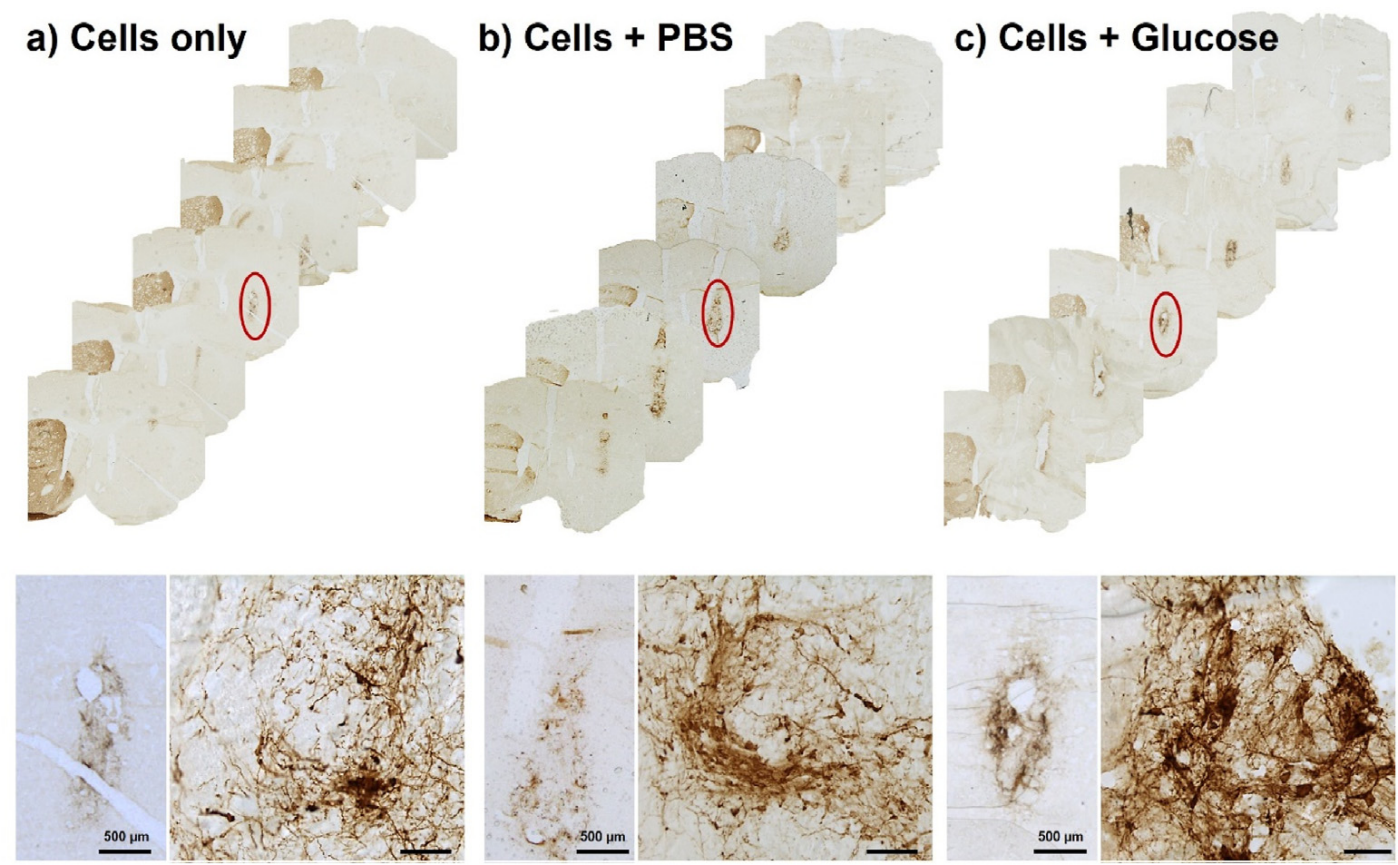
TH immunostaining identified the successful transplantation of dopaminergic neurons in each group. Representative images of coronal brain sections organised anterior to posterior, showing the unilateral 6-OHDA lesion and TH^+^ graft, with low magnification and high magnification images (taken from region marked in red) of the grafted cells either without a cannula (A), with an infusion of phosphate buffered saline (PBS) (B) or an infusion of glucose (C). Unmarked scale bars represent 50 ​μm.

Having modelled a likely transplantation scenario to investigate the time-course of oxygen and glucose depletion, we next examined the separate and combined roles played by oxygen and glucose on cell viability *in vitro* using neuronal cell types commonly studied for neural transplantation. Three hPSC lines differentiated into dopamine neuron (DA) [30] (Fig. 5), medium spiny neuron (MSN) [31] (Fig. 6), or glutamatergic projection neuron (GPN) [32] (Fig. 7) progenitor fates were analyzed, as well as human foetal ventral mesencephalon progenitors (VM) (Fig. 8). Cells were cultured in either normoxic (20 ​% oxygen) or hypoxic (0.1 ​% oxygen) conditions in combination with a gradient reduction of glucose (from 1 to 0 ​mg/mL).

**Fig. 4:**
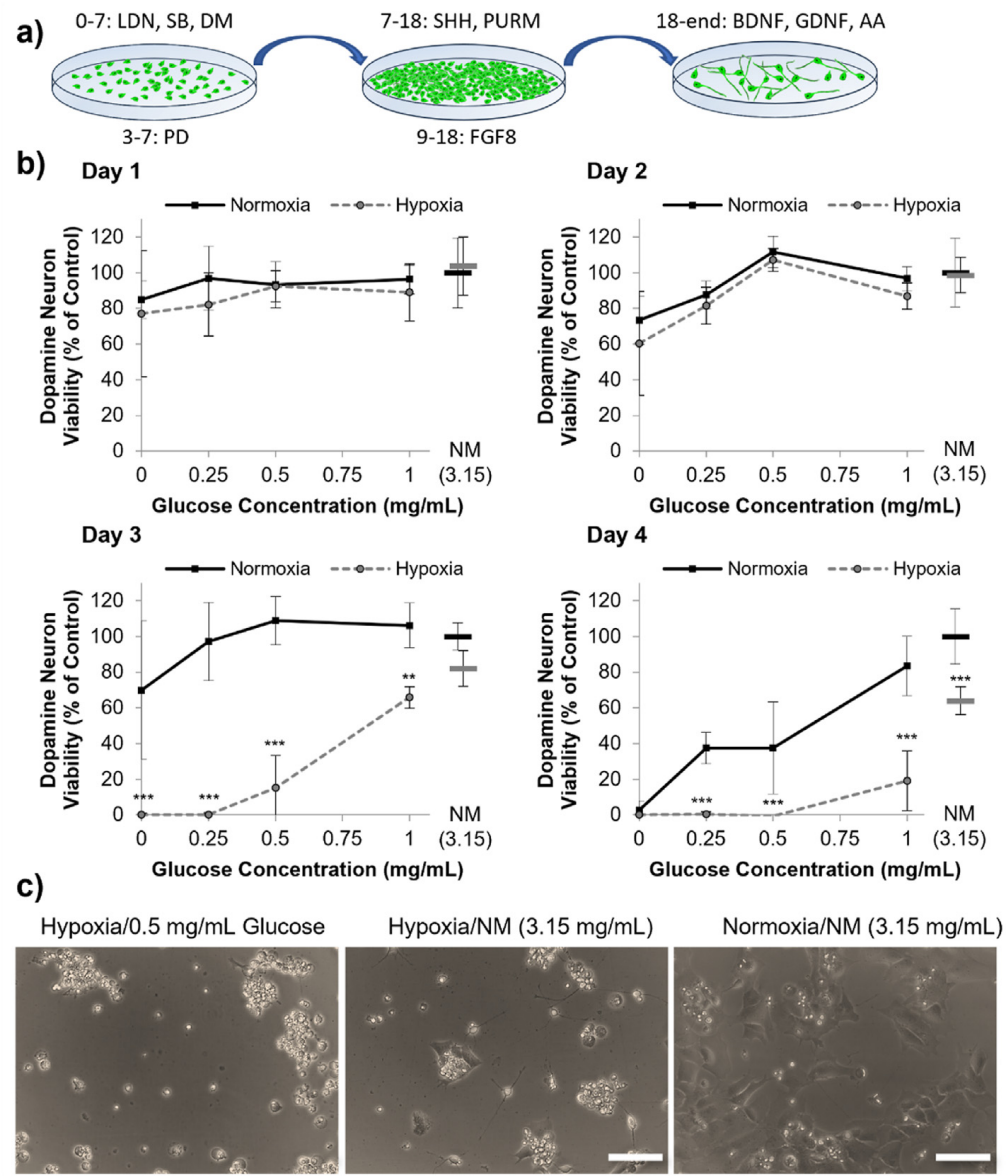
Stem cell-derived dopamine neuron progenitors lose viability after two-three days in oxygen-glucose deprivation conditions. (A) Overview of DA neuron progenitor differentiation. (B) The viability of DA neuron cultures, assessed using the PrestoBlue assay, following 1–4 days in normoxic (black, solid), or hypoxic (grey, dashed) conditions with different concentrations of glucose, where NM ​= ​normal medium containing 3.15 ​mg/mL. Data was normalized to normoxia normal media control (3.15 ​mg/mL glucose) (mean ​± ​SD, n ​= ​4). Asterisks mark cumulative effect of oxygen-glucose deprivation showing statistically significant reduced viability in hypoxia compared to normoxia for a given glucose concentration (∗∗p ​< ​0.01, ∗∗∗p ​< ​0.001) (C) Representative brightfield images of DA neurons at day 3 of culture in either hypoxic or normoxic conditions with 0.5 or 3.15 ​mg/mL glucose.

For the DA neuron progenitor cells, a paucity of glucose reduced the cell viability from day 2 [Day 2 Glucose: F4,40 ​= ​13.67, p ​< ​0.001] and hypoxia reduced the cell viability from day 3 [Day 3 Oxygen: F1,40 ​= ​177.64, p ​< ​0.001]. The combined effect of oxygen glucose deprivation became evident on day 3 as cells in glucose concentrations of 0–1 ​mg/mL, but not in normal media, lost viability dramatically in hypoxia conditions [Oxygen∗Glucose: F4,40 ​= ​10.26, p ​< ​0.001] (Fig. 4).

For MSN progenitors, the effect of glucose paucity was more pronounced as already at day 1, cell viability was higher when cultured with higher levels of glucose [Day 1 Glucose: F4,40 ​= ​27.62, p ​< ​0.001]. Overall hypoxia reduced cell viability from day 3 [Day 3 Oxygen: F1,40 ​= ​5.18, p ​< ​0.05] but even by day 4 there was no cumulative effect of combined oxygen and glucose deprivation [Oxygen∗Glucose: F4,40 ​= ​0.12, p ​> ​0.05] (Fig. 5).

Interestingly, the GPN progenitors on day 2, 3 and 4 of culture, had statistically significantly higher viability in hypoxic conditions than normoxic conditions when cultured in 3.15 ​mg/mL of glucose in the medium (*p* < 0.001) (Fig. 6b). However, the GPN progenitors lost viability in low glucose conditions rapidly from day 1 [Glucose: F4,40 ​= ​1896.51, p ​< ​0.001]. The combined effect of oxygen-glucose deprivation was also more rapid than the DA neuron progenitors, with reduced GPN progenitor viability in hypoxic conditions shown at day 2 [Oxygen∗Glucose: F4,40 ​= ​216.39, p ​< ​0.01] (Fig. 6).

Overall, these data indicate that glucose probably plays a critical role in the survival of both fetal and hPSC derived neuron progenitors. For DAs, MSNs and GPNs, concomitant hypoxia exacerbated the effect of reduced glucose, causing starkly reduced viability by day three/four, though this was not analyzed in VM culture due to limited cell supply. However, all progenitor types could survive prolonged hypoxia, without reduced survival, provided that sufficient glucose (at least 3.15 ​mg/mL) was present in the medium when the experiment commenced. This showed that glucose is the key regulator of survival in these conditions.

### *In vivo* study using osmotic minipumps to provide exogenous glucose to grafted hVM cells

*In vitro* results demonstrated that increased glucose availability can enable hVM cells to overcome temporary oxygen deprivation. An *in vivo* study was conducted to determine whether delivery of glucose to grafted hVM cells could improve their survival in the early ischemic stages post-transplantation. All 19 rats included within the study survived the duration of the experiment.

#### Glucose delivered at 0.11 ​μL per hour, failed to improve the survival of dopamine progenitor cells

**Fig. 9:**
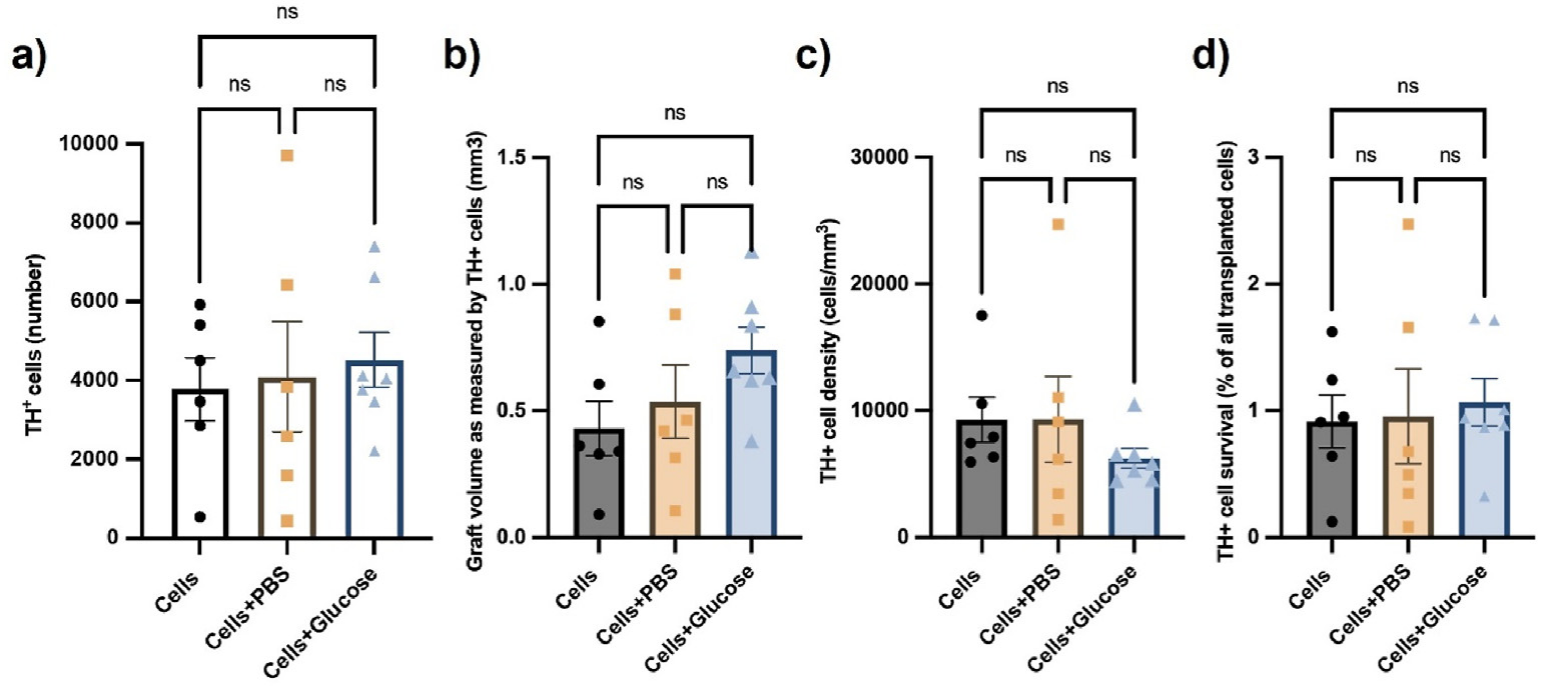
Exogenous glucose did not promote TH ​^+^ ​cell survival after 7-day post transplantation. Quantification of (A) total number of TH^+^ cells, (B) the graft volume as measured by TH^+^ cells, (C) TH^+^ cell density and (D) TH^+^ cells as a percentage of the total number of transplanted cells. TH^+^ cell survival was very similar between all groups with no statistically significant difference indicating that the amount of glucose delivered had no effect on cell survival. Data are represented as mean ​± ​SEM of n ​= ​6 rats (control and PBS groups) and 7 rats (glucose group). Statistical analysis performed with one-way ANOVA with Tukey's post-hoc analysis; ns indicates no significant differences between the groups.

The total number of TH immuno-positive dopaminergic progenitors were counted from brain sections by three independent blinded assessors and was similar across the three experimental groups (Fig. 8). The average number of TH^+^ cells counted was 3787 for the cells only group, and 4096, and 4605 for cells grafted with an osmotic minipump delivering PBS and glucose respectively (Fig. 9A) (Group: F (2, 16) ​= ​0.1467, *p* ​= ​0.8647). Despite this trend towards increased survival of dopaminergic neurons, no statistical significance was reached between the three groups, for either the TH^+^ cell count, graft volume (Fig. 9B) (Group: F (2, 16) ​= ​1.945, *p* ​= ​0.1754) or the graft density (Fig. 9C) (Group: F (2, 16) ​= ​0.7056, *p* ​= ​0.506). Dopamine cell survival was less than 1 ​% of the total cells injected across all groups with insignificant differences between groups (Fig. 9D) (Group: F (2, 16) ​= ​0.09322, *p* ​= ​0.9115). These findings conclude that exogenous glucose, provided at a rate 0.1 ​mg/day by osmotic minipumps, was not sufficient to support cell survival after 7-day post transplantation.

#### Osmotic minipump use was associated with an elevated host response and tissue damage at the graft site

**Fig. 10:**
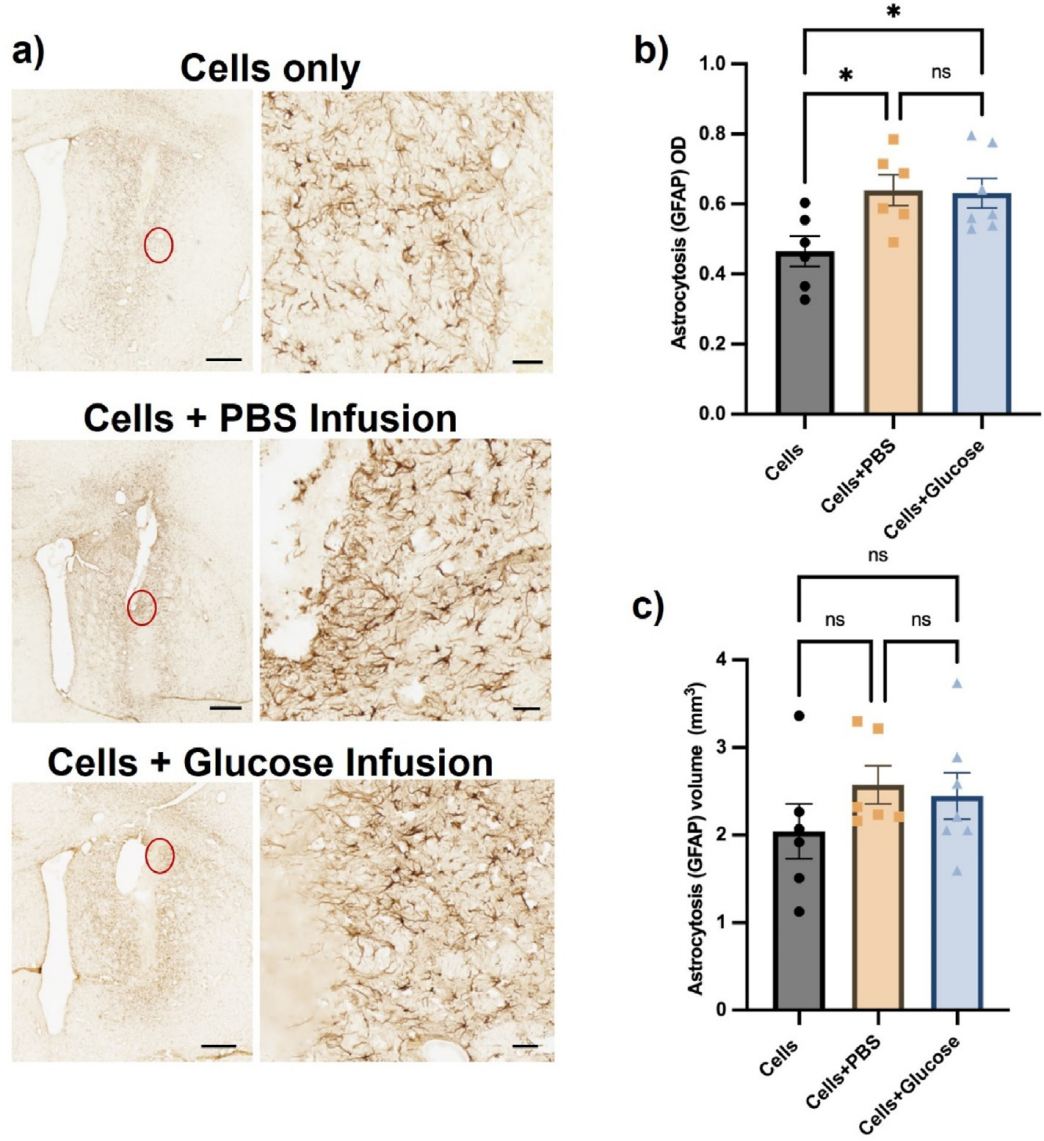
Minipump infusion increased the density of the host astrocytic response, but not the volume of astrocytosis, *in vivo* after 7-day post transplantation. (A) Representative photomicrographs of GFAP immunostaining display the elevated astrocyte response to the osmotic minipumps. Quantification of GFAP^+^ astrocytes in terms of optical density (OD) (B) and volume of striatal tissue occupied (C) showed that the infusion significantly increased the OD of the host astrocytic reaction compared to cells only group, but not the volume. Scale bars represent 500 ​μm (low magnification) or 50 ​μm (high magnification). Data are represented as mean ​± ​SEM of n ​= ​6 rats (control and PBS groups) and 7 rats (glucose group). Statistical analysis performed with one-way ANOVA with Tukey's post-hoc analysis, ∗ represents p ​< ​0.05.

**Fig. 11:**
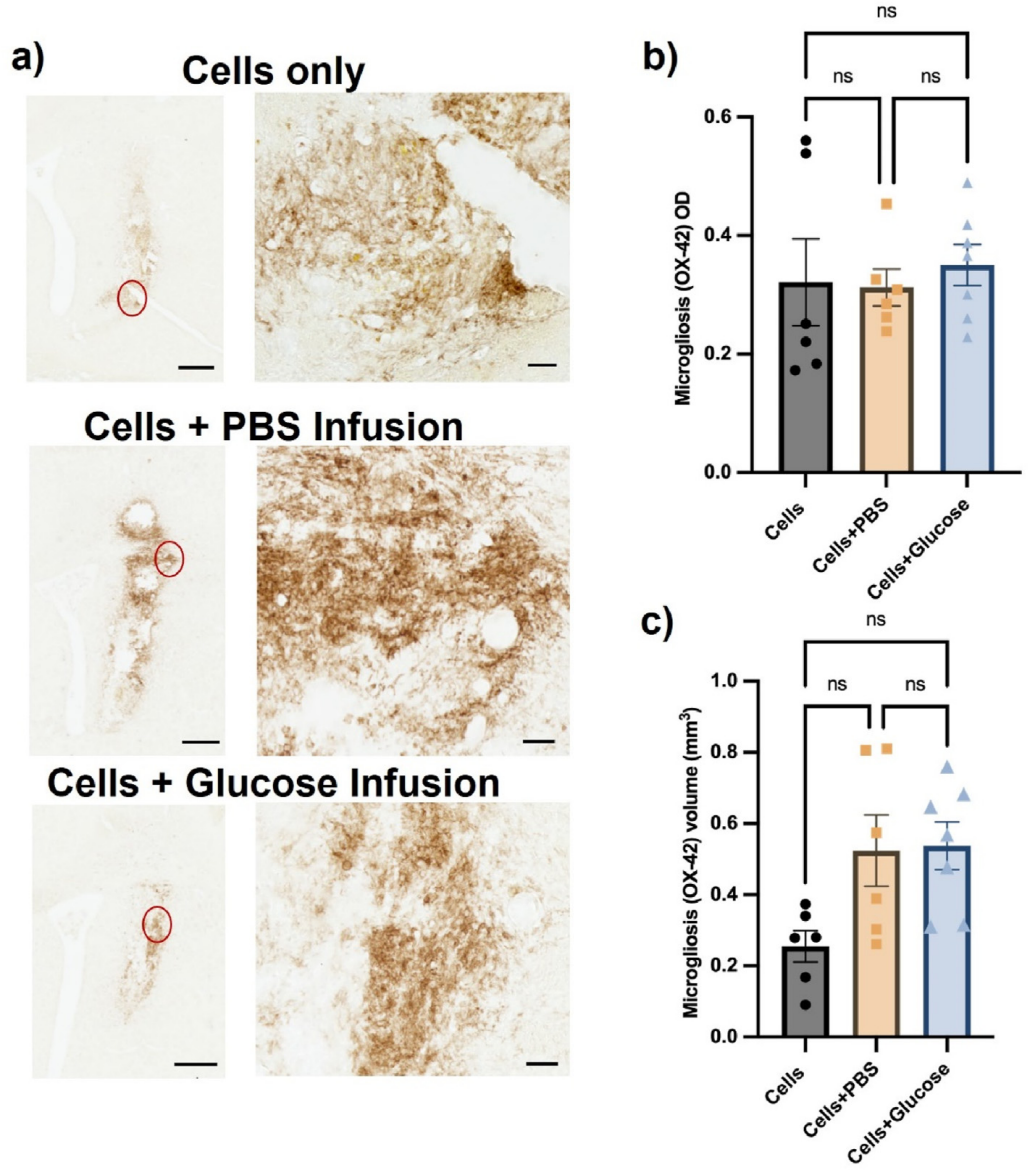
The minipump infusion had no effect on the host microglial response *in vivo* after 7-days post-transplantation. (A) Representative photomicrographs of OX-42 immunostaining display the microglia response to the graft and/or cannula. Quantification of OX-42+ microglia in terms of optical density (OD) (B) and volume of striatal tissue occupied (C) showed that the infusion did not induce any greater microglial response compared to the cells-only group. Scale bars represent 500 ​μm (low magnification) or 50 ​μm (high magnification). Data are represented as mean ​± ​SEM of n ​= ​6 rats (control and PBS groups) and 7 rats (glucose group). Statistical analysis performed with one-way ANOVA with Tukey's post-hoc analysis, where ns represents no statistically significant differences between the groups.

Inflammatory responses were assessed via analysis of the striatal astrocytic (Fig. 10A) and microglial (Fig. 11A) response surrounding the graft site/cannula. The inflammation was assessed initially by measuring the optical density of either astrocytosis (Fig. 10B) or microgliosis (Fig. 11B). The average astrocytic density showed a significant increase between the control group (0.47) and infusion groups (PBS ​= ​0.64 and glucose ​= ​0.66) with *p* ​< ​0.05 (Group: F (2, 16) ​= ​4.974, *p* ​= ​0.0209). This shows that cannulation leads to an increase in astrocytic response in the rat brains 7-day post transplantation, though this did not result in a bigger volume of the astrocyte scar (Fig. 10C) (Group: F (2, 16) ​= ​1.025, *p* ​= ​0.03812). The average microglial density and volume (Fig. 11C) was similar across the groups and none of the results showed a statistically significant difference (density, Group: F (2, 16) ​= ​0.1717, *p* ​= ​0.8438) (volume, Group: F (2, 16) ​= ​3.094, *p* ​= ​0.0731).

Critically, glucose infusion caused no elevation in the host microglial and astrocyte response when compared to PBS, indicating that any adverse response is due to the canula rather than the provision of exogenous glucose.

## Discussion

Cell death post-transplantation remains as a major limitation of cell therapy in neurodegenerative disease. Studies have demonstrated that cells experience severe hypoxia upon transplantation into the brain, and the authors suggested that this leads to their death [1]. However, we proposed herein that a paucity of glucose, or oxygen-glucose deprivation in combination, is the key driver of cell death in the early stages post-transplantation. Mathematical modelling predicted rapid oxygen and glucose depletion in grafted cells, with those furthest from the perfused periphery (i.e., cells in the centre of the graft) being worst affected. This analysis was based on previously published glucose consumption values for neurons in both normoxic and hypoxic conditions [20]. It should be noted here that their consumption experiments were performed *in vitro* and that every cell type transplanted will have differing metabolic profiles. However, this data showed that oxygen depletion is extremely rapid, and coincides with glucose exhaustion, leading to ischemia in the graft.

We recently suggested that a lack of glucose, not oxygen, plays a crucial role in graft death. For example, when mesenchymal stem cells (MSCs) were cultured in normoxic conditions without glucose for four days, their viability was reduced to less than 60 ​% [13]. In contrast, providing 1 ​mg/mL of glucose resulted in over 80 ​% viability in both anoxia and normoxia. This trend was seen herein with neuron progenitors. Moreover, Deschepper et al. established that in anoxic conditions, MSCs were able to switch to glycolysis and survive for two weeks if cultured with sufficient glucose [15]. To test the hypothesis that glucose is crucial to neuron survival, we examined neuron progenitor viability *in vitro* in normoxia or near anoxia at different glucose concentrations. Our data demonstrates that glucose deprivation, not oxygen deprivation, is the primary cause of decreased neuron survival in OGD environments, and that increased glucose availability can enable these cells to overcome temporary oxygen deprivation. Importantly, we demonstrate this effect across three different neuronal precursor cell products, all of which are in development for clinical application. Since all the neural cultures used comprised a mixed cell population [33,34], OGD could have varying effects on the different cell types present. For example, it has been reported that glial cells are more resistant to OGD than neurons [35], thus variations in the cell populations may also change their overall susceptibility to oxygen-glucose deprivation. Overall, these outcomes support the idea that inadequate glucose supply to a graft is a major contributor to cell death in the early phase post-transplantation.

We proposed that continuous glucose supplementation at the graft site could counter the predicted glucose paucity and increase graft survival in rat brains. To the best of our knowledge, this is the first *in vivo* analysis using osmotic minipumps to deliver glucose to cells transplanted to the brain. Our results suggest that exogenous glucose failed to support the survival of transplanted graft populations in the brain as no significant difference was observed between the control and glucose groups in any outcome measured (TH^+^ cell number, volume or density). Previous studies indicate that dopamine neuron survival rates after transplantation substantially vary and are typically less than 5 ​% [7,36]. Consequently, the results produced from this study are comparable to those of earlier published studies. Although this is the first study attempting to enhance the survival of grafted dopaminergic neurons using glucose, several reasons may account for the ineffectiveness of glucose supplementation in promoting the survival of grafted cells.

The first possible reason is the short duration of the study which may not have allowed sufficient time for the full maturation of dopamine progenitor cells. Since these cells usually express TH^+^ later in the maturation process [37], any potential benefits on grafted neuron maturity may not have been revealed yet. Another question is the accurate delivery of glucose to the graft. Osmotic pumps have been used in research for over 30 years as a reliable and suitable method for continuous dosing of a wide range of experimental agents [38]. They steadily release the contents of the pump using an osmotic outer layer which expands when water enters it, pushing out the contents of the pump. However, blockage of the infusion at any point during the experiment is still possible. It should be mentioned that the tubing to one of the cannulas from the glucose group was found to be bent upon extraction, consequently, the glucose in that pump was most likely not effectively delivered in that rat. Without any immunohistochemical marker of glucose, we are unable to analyse whether the grafts are effectively receiving glucose or determine the volume of tissue that it reaches.

Ensuring the appropriate amount of glucose for the graft is probably very important, but hard to estimate. According to Wohnsland et al. [20], the reported cellular glucose consumption rate of primary neurons (not dopamine progenitors) is 1.6 ​mmol/L/min in extreme hypoxia (3.91 ​× ​10^−6^ μmol/cell/day). From this data, we calculated an approximate glucose consumption rate of our graft (500,000 VM cells to be 0.35 ​mg/day of glucose). Since the rate of the osmotic minipumps was pre-set to 0.11 ​μL/h, a glucose concentration of 132 ​mg/mL would have been necessary. Our *in vitro* results showed that the lowest concentration of 0.25 ​mg/mL of glucose (equivalent to 0.025 ​mg/day) was sufficient for the survival of 25,000 VM cells, which is 0.5 mg/day for 500,000 VM transplanted cells. This would require an even higher glucose concentration of 189 ​mg/mL. However, such a concentrated solution (a viscous, almost syrup-like solution) could lead to a high risk of glucose toxicity and or/osmotic imbalance, especially to cells near to the cannula. Since high concentrations of glucose have been reported to be neurotoxic [39,40], we felt compelled to reduce the glucose concentration, though we hoped that it would still be sufficient for maintaining VM cell viability. Our decision to deliver 0.1 ​mg/day (38 ​mg/mL of glucose concentration) was seen as the optimal compromise between minimizing toxicity and providing a baseline level of glucose which we hoped would promote survival. However, this low level of glucose administered may not have been sufficient to promote survival and further study with dose escalation may shed further light on whether increased glucose would a) nourish the graft, or b) show elevated toxicity over the saline control group.

**Fig. 12:**
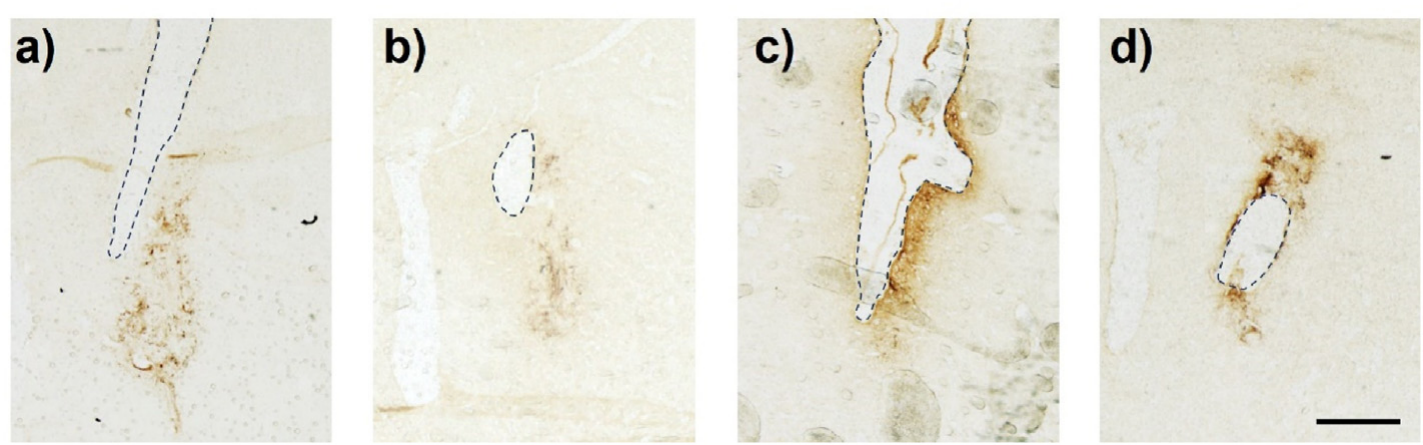
Mis-location of the cannula leads to invalid TH^+^ counting. Images show TH_+_ immunostained cells with the dashed line outlining the cannula tract of the brain infusion kit used for the osmotic minipump. The cannulas were intended to be placed adjacent to the grafted cells as shown in (A and B). However, in some animals the cannula was inside the graft, causing inaccurate TH^+^ analysis as removal of the cannula during animal sacrifice pulled out the graft with it (C, D). Scale bar represents 1 ​mm.

Further considerations include the location of the cannulas in the brain and the diffusivity of glucose through the extracellular matrix. Our goal was to insert the cannula into the striatum just above the location of the graft to maximize glucose delivery to the neuron progenitors. However, there was a variation in the cannula depth between the cannulated animals. Some of the rats had their cannula directly within the graft, leading to graft removal during the post-sacrifice decannulation prior to tissue processing (Fig. 12). This was one of the key reasons for a large disparity in the numbers of TH^+^ cells counted, as although grafted cells could clearly be visualized at the border of the cannula site, we have no idea how much of the graft was damaged upon removal of the cannula.

The microglial/astrocytic response results suggest that the insertion of a cannula and osmotic pump immediately after transplantation is largely well accepted by the host, except for an increase of the density of GFAP-positive astrocytes. This increased response is anticipated due to the increased damage caused by cannulation, which stayed in place for the full seven days. It has previously been found that increased glucose uptake in endothelial cells in hyperglycemic conditions increases the inflammatory response leading to a greater microglial response [41,42]. One positive aspect of our study is that no statistically significant difference in host microglial response was observed between the cannulated groups, emphasizing that the glucose did not increase the microglial response in the graft site. Subsequently, increased dosages could be explored in future studies.

The concept of direct infusion into the brain through cannulation has been extensively explored, including in the recent glial cell line-derived neurotrophic factor (GDNF) delivery trial [43]. However, the osmotic minipumps here revealed a limitation of this strategy in terms of the tissue damage at the graft site. From a clinical viewpoint, the TRANSEURO trial for cell transplantation in PD employed five tracts per hemisphere, and each tract comprised eight deposits of cells [8]. Therefore, a single cannula would be unable to perfuse all the tracts, and the delivery might be limited only to the proximal deposits. It is therefore essential to develop alternative methods of glucose delivery to fully address this problem of graft oxygen-glucose deprivation.

One approach could be the use of glucose-releasing biomaterials, an emerging field in which a few systems are described in the literature [[44], [45], [46], [47], [48]]. However, these typically display a short release duration or are not small enough to be co-administered with cells into the brain. Our modelling and *in vitro* data, coupled with the cannula technique confounding the *in vivo* analysis, highlight the importance of formulating a delivery system that achieves a prolonged release of glucose, at least until a functioning blood supply is completed throughout the graft.

In conclusion, most cells transplanted into the brain die before they can produce a functional effect, reducing the potential efficacy of cell replacement therapies for neurodegenerative disease. There is increasing evidence to suggest that glucose deficiency is an important factor in cell survival post-transplantation. This is the first *in vivo* study that explores the possibility that a paucity of glucose is the crucial reason for dopamine neuron progenitor cell death post-transplantation in a rat model of PD. We did not show improved survival through the provision of exogenous glucose to the graft, though the amount delivered may be far lower than that required to sustain graft viability. Furthermore, efficient and less invasive glucose delivery may well be critical, highlighting the need to explore alternative glucose delivery systems. The development of injectable glucose delivery systems, which control the release in a sustained manner, may well help prove whether glucose paucity is a key factor in graft survival post-transplantation to the brain.

## Author Contributions

Conceptualization (SAR, MJL, BN); Formal Analysis (AH, SAR, OJMB, TEW, BN); Funding Acquisition (MJL, BN); Investigation (AH, SAR, OJMB, RH, SVP, TO, MF, MA, ELL, TEW, MJL, BN); Methodology (RH, SVP, AER, ELL, MJL, BN); Project Administration (BN); Resources (AER, TEW, MJL, BN); Supervision (RH, AER, TEW, BN); Validation (AH, SAR, OJMB); Visualization (AH, SAR, OJMB, BN); Writing - Original Draft (AH, SAR, OJMB, BN); and Writing - Review & Editing (AH, SAR, OJMB, BN).

## Data Availability

Data will be made available on request.

## Declaration of competing interest

The authors declare the following financial interests/personal relationships which may be considered as potential competing interests: Ben Newland reports financial support was provided by Cardiff University. If there are other authors, they declare that they have no known competing financial interests or personal relationships that could have appeared to influence the work reported in this paper.
