# Supplementary material for: Graft ischemia post cell transplantation to the brain: Glucose deprivation as the primary driver of rapid cell death
Source: Neurotherapeutics. 2025 Jan 9;22(2):e00518. doi: 10.1016/j.neurot.2024.e00518 (PMC12014406; doi:10.1016/j.neurot.2024.e00518)
Supplement: Multimedia component 1 [file mmc1.docx]

**Graft Ischemia Post Cell Transplantation to the brain: Glucose Deprivation as the Primary Driver of Rapid Cell Death**

Abrar Hakami^1,2^, Sebastiano Antonio Rizzo^1^, Oliver J.M. Bartley^3^, Rachel Hills^3^, Sophie V. Precious^3^, Timothy Ostler^4^, Marija Fjodorova^5^, Majed Alghamdi^1,2^, Anne E. Rosser^3,5,6^, Emma L. Lane^1^, Thomas E. Woolley^4^, Mariah J. Lelos^3^, Ben Newland^1,7*^

**Supplementary Information**


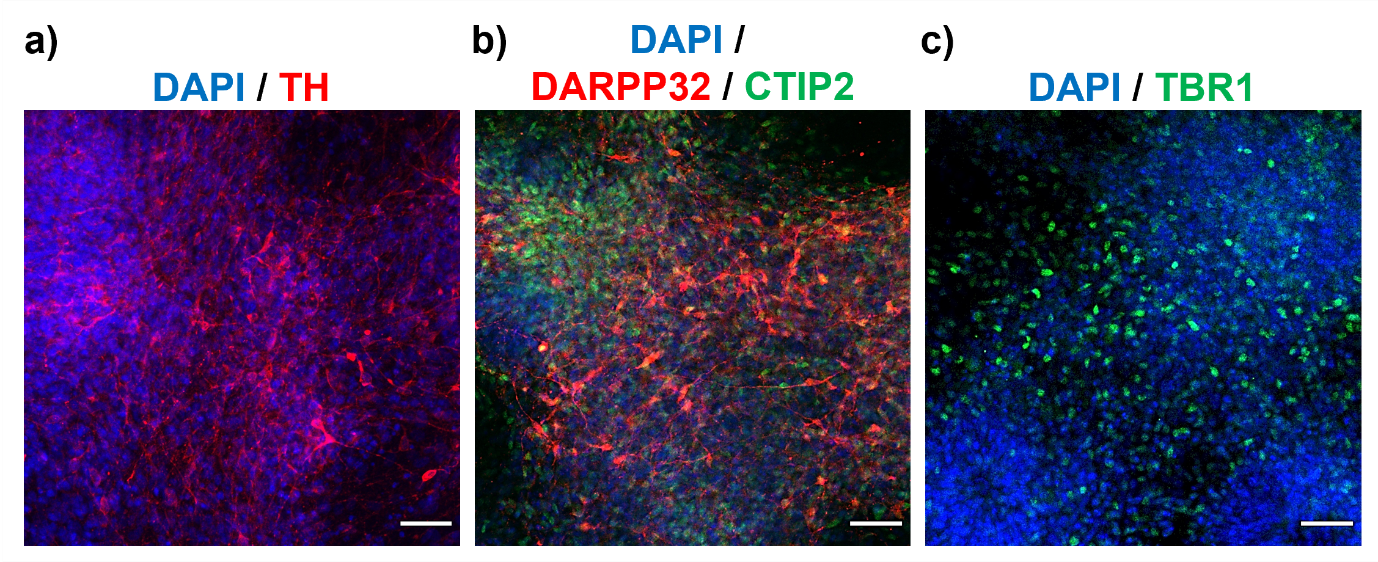


SI Figure S1 Neuron progenitor characterization. Immunocytochemical analysis of the three neuron types at 40 days in vitro, with a) dopamine neurons visualized via tyrosine hydroxylase (TH) immunostaining, b) medium spiney neurons via dopamine- and cAMP-regulated neuronal phosphoprotein (DARPP-32) and COUP-TF-interacting protein 2 (CTIP2), and c) glutamatergic projection neurons via T-box, brain, 1 (TBR1). The full quantitative characterization of these cells is reported in a previous publication [1].

**References**

1. Fjodorova M, Noakes Z, De La Fuente DC, Errington AC, Li M. Dysfunction of cAMP–protein kinase A–calcium signaling axis in striatal medium spiny neurons: A role in schizophrenia and Huntington’s disease neuropathology. Biological Psychiatry Global Open Science. 2023;3(3):418-29.
